# Thiolated Chitosan Microneedle Patch of Levosulpiride from Fabrication, Characterization to Bioavailability Enhancement Approach

**DOI:** 10.3390/polym14030415

**Published:** 2022-01-20

**Authors:** Rukhshanda Habib, Abul Kalam Azad, Muhammad Akhlaq, Fakhria A. Al-Joufi, Gul Shahnaz, Hanan R. H. Mohamed, Muhammad Naeem, Abdulraheem S. A. Almalki, Junaid Asghar, Aamir Jalil, Mohamed M. Abdel-Daim

**Affiliations:** 1 Faculty of Pharmacy, Gomal University, Dera Ismail Khan 29050, Pakistan; rukhshandahabib.isb@gmail.com (R.H.); dr.akhlaq@gu.edu.pk (M.A.); junaid.asghar@gu.edu.pk (J.A.); 2 Department of Pharmacology, University of Oxford, Mansfield Rd., Oxford OX1 3QT, UK; 3 Department of Pharmacy, Quaid-I-Azam University, Islamabad 45320, Pakistan; gshahnaz@qau.edu.pk; 4 Department of Biotechnology, Quaid-I-Azam University, Islamabad 45320, Pakistan; mnaeem@qau.edu.pk; 5 Pharmaceutical Technology Unit, Faculty of Pharmacy, AIMST University, Bedong 08100, Kedah, Malaysia; 6 Department of Pharmacology, College of Pharmacy, Jouf University, Skaka 72341, Saudi Arabia; faaljoufi@ju.edu.sa; 7 Zoology Department, Faculty of Science, Cairo University, Giza 12613, Egypt; hananeeyra@gmail.com; 8 Department of Chemistry, Faculty of Science, Taif University, P.O. Box 11099, Taif 21974, Saudi Arabia; almalki.a@tu.edu.sa; 9 Faculty of Pharmacy, Bahauddin Zakariya University, Multan 60800, Pakistan; aamirs.jalil@gmail.com; 10 Department of Pharmaceutical Sciences, Pharmacy Program, Batterjee Medical College, P.O. Box 6231, Jeddah 21442, Saudi Arabia; 11 Pharmacology Department, Faculty of Veterinary Medicine, Suez Canal University, Ismailia 41522, Egypt

**Keywords:** levosulpiride, thiolated chitosan, transdermal delivery, microneedle patches, bioavailability enhancement, in-vitro evaluation, in-vivo evaluation

## Abstract

In this study, a first attempt has been made to deliver levosulpiride transdermally through a thiolated chitosan microneedle patch (TC-MNP). Levosulpiride is slowly and weakly absorbed from the gastrointestinal tract with an oral bioavailability of less than 25% and short half-life of about 6 h. In order to enhance its bioavailability, levosulpiride-loaded thiolated chitosan microneedle patches (LS-TC-MNPs) were fabricated. Firstly, thiolated chitosan was synthesized and characterized by nuclear magnetic resonance (^1^HNMR) spectroscopy, attenuated total reflectance-Fourier transform infrared (ATR-FTIR) spectroscopy, differential scanning calorimetry (DSC), and X-ray diffraction (XRD). Thiolated chitosan has been used in different drug delivery systems; herein, thiolated chitosan has been used for the transdermal delivery of LS. LS-TC-MNPs were fabricated from different concentrations of thiolated chitosan solution. Furthermore, the levosulpiride-loaded thiolated chitosan microneedle patch (LS-TC-MNP) was characterized by FTIR spectroscopic analysis, scanning electron microscopy (SEM) study, penetration ability, tensile strength, moisture content, patch thickness, and elongation test. LS-TC-MNP fabricated with 3% thiolated chitosan solution was found to have the best tensile strength, moisture content, patch thickness, elongation, drug-loading efficiency, and drug content. Thiolated chitosan is biodegradable, nontoxic and has good absorption and swelling in the skin. LS-TC-MNP-3 consists of 100 needles in 10 rows each with 10 needles. The length of each microneedle was 575 μm; they were pyramidal in shape, with sharp pointed ends and a base diameter of 200 µm. The microneedle patch (LS-TC-MNP-3) resulted in-vitro drug release of 65% up to 48 h, ex vivo permeation of 63.6%, with good skin biocompatibility and enhanced in-vivo pharmacokinetics (AUC = 986 µg/mL·h, Cmax = 24.5 µg/mL) as compared to oral LS dispersion (AUC = 3.2 µg/mL·h, Cmax = 0.5 µg/mL). Based on the above results, LS-TC-MNP-3 seems to be a promising strategy for enhancing the bioavailability of levosulpiride.

## 1. Introduction

Levosulpiride (LS) is [5-(amino-sulfonyl)-N-[(1-ethyl-2-pyrrolidinyl)methyl]-2-methoxy benzamide]. It is the levo-enantiomer of racemic sulpiride. It has several pharmacological properties, including antipsychotic and antidepressant action and also effective against ulcer [1]. It is believed that these activities are mediated mainly by the selective inhibition of the dopamine D_2_ receptor in the trigger zone, which is effective both in the gastrointestinal tract and central nervous system [2,3]. Orally, levosulpiride is slowly and weakly absorbed in the gastrointestinal tract (GIT) with a half-life of about 6 h and oral bioavailability of less than 25% [4]. Levosulpiride is a biopharmaceutical classification system (BCS) class IV drug; it has low water solubility and low permeability [5]. It is not readily metabolized, as 70–90% of the intravenous dose and 15–25% of oral dose are excreted unchanged in the urine [6]. As a result, levosulpiride is clinically used in higher doses (300 to 600 mg orally) for the treatment of psychopathology of senes-cence, schizophrenia, and depression. Additionally, the drug is useful in lower doses (75 mg orally) for the treatment of irritable colon syndrome and duodenal or gastric ulcer related to psychosomatic stress [7]. Previously, several attempts, including self-micro-emulsifying carriers [8], micro-sponges [9], solid dispersion [10], micro-capsules [11], and solid lipid nanoparticles [12] has been done to overcome LS oral complications. However, the outcomes were inadequate, as these attempts mainly ad-dresses the issue of poor aqueous solubility. Hence, other platforms are needed to overcome the oral delivery challenges and enhance the pharmacological efficacy of LS.

A transdermal drug delivery system (TDDS), compared to intramuscular and intravenous routes, is a non-invasive and painless drug delivery system. A TDDS is oftently used drug delivery route for increasing the bioavailability of BCS class IV drugs. Patients can self-administer TDDS easily and quickly, thus achieving higher patient compliance [13,14,15] and preventing first-pass metabolism by the liver [16,17]. The skin, especially the upper stratum corneum, is the main barrier to transdermal drug delivery [18]. Microneedles are minimally noninvasive devices capable of penetrating the stratum corneum to overcome barrier properties [19,20,21,22]. The biocompatibility and me-chanical properties of the materials selected for microneedle (MN) fabrication are critical for MN performance. The most important properties for MN material selection are lower production costs and higher mechanical strength [23]. Depending on the composition of the material, MNs can be divided into polymeric, inorganic or metal microneedles. Due to the unique advantages of polymers, such as excellent biocompatibility, high drug loading, the easy and inexpensive production process of polymeric MNs, these are the highly desirable transdermal drug delivery systems [24]. Choosing the right material and geometry plays a vital role in the design of the MN array.

Most water-soluble polymers (polysaccharides) are mechanically weaker than silicon or non-soluble materials. Chitosan has shown interesting properties for drug delivery and biomedical applications, but its use is limited by solubility issues, pH-dependent changes in electrostatic charge, and low mucosal adhesion [25]. These issues could be solved by thiolation of chitosan; thiolated polymers have been used in a variety of applications, including drug delivery, tissue engineering, textile industry, water purification, and many other biomedical applications [26]. As thiolated chitosan has excellent mechanical strength and greater water uptake ability due to thiol moieties, thiolated chitosan (TC) was selected as the microneedle fabrication material in this study. Therefore, the aim of this study was focused on the synthesis of thiolated chitosan, thereafter, fabrication of microneedles from synthesized thiolated chitosan. The investigation of the role of a thiolated chitosan microneedles patch for enhanced transdermal drug delivery, in-vitro, ex-vivo, in-vivo pharmacokinetic, and improvede bioavailability of levosulpiride.

## 2. Materials and Methods

### 2.1. Materials

Levosulpiride (Biolabs, Islamabad, Pakistan); chitosan (low molecular weight, degree of deacetylation 75–85%) (Merck, Darmstadt, Germany); thioglycolic acid (TGA 99%) (Merck, Darmstadt, Germany); 1-ethyl-3-(3-dimethylamino propyl) carbodiimide hydrochloride (EDAC) (Merck, Darmstadt, Germany); 5,5-dithiobis (2-nitrobenzoic acid) (Ellmans reagent) (Merck, Darmstadt, Germany); 2,4,6-trinitrobenzene sulfonic acid (TNBS) (Merck, Darmstadt, Germany); potassium dihydrogen phosphate (Merck, Darmstadt, Germany); disodium dihydrogen phosphate (Merck, Darmstadt, Germany); acetonitrile (Merck, Darmstadt, Germany); methanol (Merck, Darmstadt, Germany); hydroxylamine (Merck, Darmstadt, Germany); sodium hydroxide (Merck, Darmstadt, Germany); sodium chloride (Merck, Darmstadt, Germany); dialysis membrane (cut-off value 12KD); glacial acetic acid (Merck, Darmstadt, Germany).

### 2.2. Synthesis and Characterization of Thiolated Chitosan

Briefly, 1% (*w*/*v*) chitosan was dissolved in acetic acid solution 1% (*v*/*v*). Then, thioglycolic acid (TGA) 500 mg was added to the chitosan solution; subsequently, 1-ethyl-3-(3-dimethylamino propyl) carbodiimide hydrochloride (EDAC) was added as a coupling reagent at a concentration of 100 mM. The addition of EDAC is necessary to stimulate the carboxylic acid group of TGA. Then, the mixture was stirred for 5 h, and 10 M NaOH was added to adjust the pH to 5.5. The thiolated polymer was dialyzed by membrane tubing with a MW cut-off of 12–14 kDa in order to remove unbound sulfhydryl moieties; the mixture was dialyzed five times at 10 °C in the dark for three days. The dialyses were performed, once against 5 mM HCl (in 5 L of deionized water); then, to break the ionic interactions between negatively charged sulfhydryl moieties and positively charged polymer, the mixture was dialyzed 2 times against 5 mM HCl and NaCl 1% (*w*/*v*). Lastly, to adjust the pH of the polymer, it was dialyzed twice against 1 mM HCl. After polymerization, dialyzed polymer was stored at 4°C [27].

#### 2.2.1. Quantitation of Primary Amine and Thiol Content

The colorimetric 2,4,6-trinitrobenzene sulfonic acid (TNBS) assay was performed to quantify the primary amine functional groups of thiolated chitosan. Briefly, 0.5 mg polymer was dissolved in a solution of 500 μL (0.5%, *w*/*v*) NaCl. Afterwards, the mixture was incubated for 30 min at 25°C. To every hydrated aliquot, 500 μL (0.1%, *w*/*v*) of TNBS containing NaHCO_3_ (4%, *w*/*v*) was added. Absorbance was checked with a microtiter plate reader (Molecular Devices, San Jose, CA, USA) at 410 nm after incubation of solution at 37 °C for 3 h and centrifugation (33,527× *g*; 4 °C; 5 min). Calculations were made with L-cysteine HCl standards.

Ellman’s reagent was used to quantify the degree of conjugation of thiol groups in thiolated chitosan (TC). Briefly, 0.5 mg of thiolated chitosan was hydrated with 500 uL (0.5 M, pH 8.0) phosphate buffer. Then, to every single aliquot, 500 µL of Ellman’s reagent (3 mg in 10 mL of phosphate buffer 0.5 M) was added. After incubation for 3 h at room temperature, a microtiter plate reader (Molecular Devices, San Jose, CA, USA) at 410 nm was used to quantify the thiol content, L-cysteine standards were used for calculations [28].

After the polymer modifications, the extent of disulfide bond formation was quantified, as reported earlier [28]. Tris buffer (pH 6.8; 0.05 M) 1 mL was added to a 15 mL Falcon tube and then 0.5 mg TC was added and allowed to swell. After incubation for 30 min at room temperature, sodium borohydride solution 4% (*w*/*v*) 1 mL was added dropwise into the above reaction mixtures. The samples were then mixed for 3 h at 37 °C. Hereafter, a solution of 5 M HCl (200 μL) was added to quench the mixture.

#### 2.2.2. Nuclear Magnetic Resonance (^1^HNMR)

Modification of chitosan to thiolated chitosan was characterized by ^1^HNMR (H NMR; 500 MHz; Varian Medical Systems, Inc., Palo Alto, CA, USA) spectroscopy [29].

#### 2.2.3. Attenuated Total Reflectance-Fourier Transform Infrared (FTIR) Spectroscopy

Functional groups of chitosan and modified chitosan and the compatibility of LS with TC in LS-TC-MNP, was investigated by FTIR. FTIR spectroscopy was performed over the range of 4000–400 cm^−1^ by using an FTIR spectrophotometer (Bruker, Billerica, Ma, USA) [30].

#### 2.2.4. Differential Scanning Calorimetry (DSC)

DSC was performed by using SD Q600 (TA Instruments, Lukens, New Castle, DE, USA) in the range of 25–300 °C, with heat flow rate set at 10 °C per min and nitrogen as purging gas [30].

#### 2.2.5. X-ray Diffraction (XRD)

XRD characterization was performed by a D2 Phaser (Bruker, Billerica, Massachusetts, USA) over the 2θ range of 0–80° at a scan rate of 0.02 °/min [30].

#### 2.2.6. Scanning Electron Microscopy (SEM) Study

The SEM analysis was performed by carefully placing the LS-TC-MNP on a carbon-coated copper mesh. In order to obtain a better comparison, a sputter coater (Denton, Desk V HP) was used, operated under vacuum at 40 mA for 15 s and the dried sample was gold plated. The sample was then visualized using scanning electron microscope (JEOL JSM 6500F SEM, Tokyo, Japan). SEM was used to visualize the surface and dimensions of LS-TC-MNP (needle height, needle base, and needle distance) [31].

### 2.3. Fabrication of Levosulpiride Loaded Thiolated Chitosan Microneedle Patch (LS-TC-MNP)

Thiolated chitosan microneedle patch was fabricated by adding different concentrations of thiolated chitosan solution into a PDMS mold. Thiolated chitosan solution (500 mg) containing (25 mg) levosulpiride was casted on the mold to form a single layer. Then mold was placed in a 50 mL flat-bottom centrifuge tube and centrifuged at 3000 rpm for 30 min. The centrifugation step was repeated 4 times, for a total time of 2 h. In order to fabricate microneedle patch base, a second layer of thiolated chitosan solution was added without levosulpiride. The second layer would be on the surface of skin and does not penetrate into the skin. Finally, the mold was again placed inside a 50 mL test tube and oven dried at 28 °C for three days without a stopper. Each LS-TC-MNP contained 25 mg of levosulpiride. Tweezers were used to carefully remove the microneedle mold from the test tube and then LS-TC-MNP was detached from mold using a heated scalpel [32].

### 2.4. Tensile Strength

The tensile strength of LS-TC-MNP was measured by using the method of Khan et al. [33]. The LS-TC-MNP was placed between the jaws of auto tensile tester (Suzhou Tophung Machinery Equipment Co., Ltd., Jiangsu, China) until the LS-TC-MNP split in two parts. The tensile strength at breaking point was calculated by using Equation (1).

(1) $$\mathrm{Tensile}\;\mathrm{strength}\;\left(\frac{N}{{\mathrm{mm}}^{2}}\right)=\mathrm{breaking}\;\mathrm{force}\;\left(N\right)\;\mathrm{sectional}\;\mathrm{area}\;\mathrm{of}\;\mathrm{sample}\;\left({\mathrm{mm}}^{2}\right)$$

### 2.5. Moisture Content

The moisture content of the LS-TC-MNP was measured according to previously developed method [34]. The percent water content of LS-TC-MNP was determined with a Q500 Thermo Gravimetric Analyzer (TA Instruments, Elstree, Hertfordshire, UK). Samples were heated from ambient temperature to 600 °C at a heating rate of 10 °C min^−1^. The data from thermogravimetric analysis experiments were analyzed with TA Instruments Universal Analysis 2000 software, version 4.4A (TA Instruments, Elstree, Hertfordshire, UK).

### 2.6. Thickness

A digital absolute micrometer (Mitutoyo, Kawasaki, Japan) was used to check the average thickness of LS-TC-MNP at different points. Firstly, thickness of the glass slide was measured and then the LS-TC-MNP was placed between the two glass slides and the micrometer measurement was repeated. The thickness of the LS-TC-MNP was obtained by subtracting the thickness of the glass slide [35].

### 2.7. Elongation

The percentage elongation of the LS-TC-MNP was measured until it was divided into two parts. An auto tensile testing machine was used to measure the starting and ending length of the LS-TC-MNP and the elongation at breaking point was calculated using Equation (2) [33].

(2) $$\mathrm{Percentage}\;\mathrm{enlongation}=\frac{\mathrm{Final}\;\mathrm{length}\;\mathrm{of}\;\mathrm{patch}\;\mathrm{at}\;\mathrm{breaking}\;\mathrm{point}\;}{\mathrm{Initial}\;\mathrm{length}\;\mathrm{of}\;\mathrm{patch}}\times 100$$

### 2.8. Penetration Ability

Parafilm-M was used to evaluate the penetration ability of LS-TC-MNP [36]. Parafilm-M was folded to achieve 8 layers with a thickness of about 1 mm. The film was placed on a flat surface and LS-TC-MNP was forced manually on it, for 30 s. The LS-TC-MNP was removed from the parafilm-M. Parafilm-M was unfolded and the number of holes was checked in each layer with the help of microscope. The shape of the microneedles before and after insertion was checked under a microscope and measurements were made with a micrometer.

### 2.9. Drug Loading Efficiency

The LS-TC-MNP was dissolved in methanol: water mixture. Then, it was diluted with sufficient quantity of mobile phase and was analyzed by HPLC according to the previously developed HPLC method by our research group [37]. The loading efficiency of LS in the LS-TC-MNP was then calculated using the Equation (3).

(3) $$\mathrm{Amount}\;\mathrm{of}\;\mathrm{drug}\;\mathrm{loaded}=\left(C_{o}\times Volume\right)-C_{1}\times \left(volume-\frac{∆M}{\rho }\right)$$

where *C*_o_ is the concentration of the solution prior to addition MN patches, ΔM is the difference in mass of the MN patch before and after loading, *C*_1_ is the concentration of the solution after addition to MN patches, and ρ is the density of water [38].

Drug loading was calculated by Equation (4).

(4) $$\mathrm{Loading}\;\mathrm{Content}\;=\frac{Total\;amount\;of\;drug\;added-Amount\;of\;unentrapped\;drug}{Total\;mass\;of\;polymer}\times 100$$

### 2.10. In-Vitro Drug Release Studies

A modified Franz diffusion cell apparatus was used to check the in vitro release of LS from LS-TC-MNP. LS-TC-MNP (with 25 mg of LS) was attached to the Parafilm-M and placed between the donor and receptor chambers of Franz diffusion cell. The sides of the Franz cell were sealed with clamps and petroleum jelly. The LS-TC-MNP was hydrated with phosphate buffer (pH 7.4). The receptor compartment was filled with 10 mL of phosphate buffer (pH 7.4) and kept at 37 ± 1 °C during the entire study, with water circulation system. After predetermined time intervals (1, 2, 4, 6, 8, 10, 12, 16, 20, 24, 30, 36, and 48 h), 0.5 mL samples were removed from the arm of the receptor compartment and replaced with an equal volume of fresh phosphate buffer (pH 7.4) kept at 37 ± 1 °C. As reported earlier, samples were then analyzed by HPLC [37]. The in-vitro release profile was then subjected to mathematical modeling by using free DD Solver software (Microsoft Excel add-in).

### 2.11. Ex-Vivo Permeation Study

The ex-vivo skin permeation study was performed on mouse skin samples. Mouse skin was shaved, and remaining hairs were carefully removed by depilatory cream. LS-TC-MNP (LS = 25 mg) was firmly attached to the skin sample and hydrated with phosphate buffer (20 µL, pH 7.4). Then, this skin was placed between the donor and receptor chamber of the Franz diffusion cell. The system was maintained at 37 ± 1 °C during the entire study. The receptor chamber was filled with 10 mL of phosphate buffer (pH 7.4). After predetermined time intervals (1, 2, 3, 4, 6, 8, 12, 16, and 24 h), 500 µL of sample was drained and refilled with fresh buffer. The samples were then analyzed by HPLC [37].

### 2.12. Skin Distribution Study

After a 24 h ex-vivo permeation study, the skin was removed from the Franz diffusion cell and homogenized with a tissue homogenizer. Homogenate was soaked in methanol under stirring overnight to extract LS. The extract was filtered through 0.45 syringe filter and analyzed by HPLC [37].

### 2.13. In-Vivo Tolerance and Safety Study

The standard Draize skin irritation test was performed to measure erythema and edema, after removal of LS-TC-MNP [39]. Mice weighing 24 ± 5 g were anesthetized using gas anesthesia of isoflurane in oxygen (2–4% (*v*/*v*)). The hairs of each mouse were removed from the back area with a professional pet clipper, 24 h prior to the study. Before insertion of LS-TC-MNP, the application area of each animal was monitored for any signs of inflammation. A visual scoring system was employed to assess the intensity of erythema. According to the Draize skin irritation test, erythema scores were recorded at 1, 6, 24, and 48 h after application. These were compared with control animals (no LS-TC-MNP). Primary irritation index (PII) was calculated by previously reported method [40].

### 2.14. In-Vivo Study

In in-vivo study, the average mouse weight was 24 ± 5 g. Mice were acclimated for 7 days prior to the study and fasted for 24 h before the study. All in-vivo experiments were approved by the ethical committee, Faculty of Pharmacy, Gomal University (No: 10/ERB/GU). The mice were anesthetized under gas anesthesia (i.e., isoflurane in oxygen (2–4% (*v*/*v*))). Bulk hairs were removed with an electrical clipper and then a depilatory cream was applied to remove the remaining hair that could interfere with the insertion of LS-TC-MNP [41,42]. Hairs were removed from the back area of mouse at 4 h prior to experiment. Mice were divided into two groups (n = 16 per group (total: 32)); oral (given the oral LS dispersion) and transdermal (LS-TC-MNP inserted in skin). Mice in the oral group received an oral LS dispersion of (200 mg/kg). The transdermal group was the LS-TC-MNP treatment group, in which each mouse was treated with (LS = 25 mg) LS-TC-MNP. To facilitate the insertion of LS-TC-MNP, the mice were anesthetized by gas anesthesia. The mice skin was pinched, and LS-TC-MNP attached to self-adhesive margins was inserted with firm finger pressure onto the back of each mouse. Blood samples were withdrawn by bleeding the tail vein at predetermined time intervals. The study plan was 16 mice per group and a maximum of (n = 3) blood samples was withdrawn from each mouse. Each mouse was bled twice a day. After 1 and 4 h, blood was withdrawn from the first 4 mice; the next 4 mice were bled after 2 and 6 h; the next four mice at 8 and 24 h; the remaining 4 mice at 12 h; and all mice (n = 16) at 48 h. The area under the curve and other pharmacokinetic parameters were measured by using Pk solver software (Microsoft Excel add-in programm).

### 2.15. Data Analysis

Statistical analysis of results was performed by one-way analysis of variance (ANOVA) and Mann–Whitney U-test; *p*-value < 0.05 was considered statistically significant. All data were expressed as mean ± standard deviation (SD) and all experiments were repeated at least three times.

## 3. Results and Discussion

### 3.1. Synthesis and Characterization of Thiolated Chitosan (TC)

**Figure 1:**
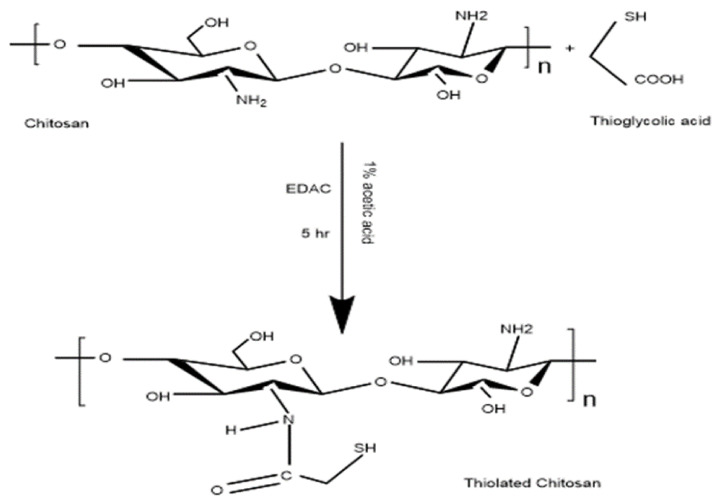
Reaction scheme for thiolation of chitosan by formation of covalent bond between thioglycolic acid and chitosan via EDAC coupling.

Thiolated chitosan was synthesized by the covalent linkage between chitosan and thioglycolic acid. During the reaction, an amide bond is formed between the carboxylate groups of the sulfhydryl moiety and the amino group of chitosan (Figure 1) [28]. Chitosan was successfully modified into thiolated chitosan (TC) by 1-ethyl-3-(3-dimethylamino propyl) carbodiimide hydrochloride (EDAC) coupling, as shown in Figure 1. The lyophilized TC appears as a white fibrous material. The number of sulfhydryl groups attached to chitosan were 448 ± 23 µmol/g polymer and the number of disulfide bonds per gram of TC were 158 ± 37 µmol. The thiol group was successfully immobilized on the chitosan backbone by the carbodiimide chemical method using EDAC to generate thiolated chitosan [43]. The sulfhydryl groups and disulfide bonds were quantified, which confirmed the successful synthesis of the thiolated polymer.

### 3.2. Nuclear Magnetic Resonance (^1^HNMR) Spectroscopy

**Figure 2:**
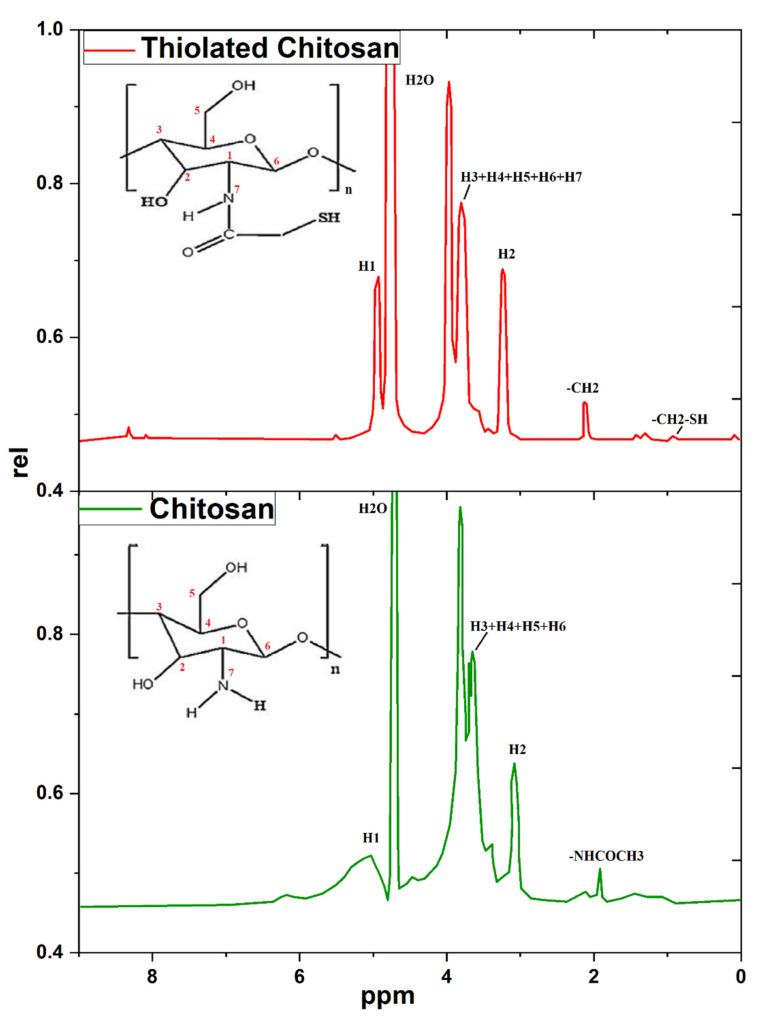
Nuclear magnetic resonance (^1^HNMR) spectra of chitosan and thiolated chitosan synthesized by covalent bond formation between thioglycolic acid and chitosan via EDAC coupling.

The ^1^HNMR spectra of chitosan in Figure 2 show a small peak at 1.79 ppm, attributable to the −CH_3_ of the N-acetyl glucosamine residue. A peak at 3 ppm is assigned to the H2 of N-acetyl glucosamine, and the peaks from 3.56–3.74 ppm correspond to the H3, H4, H5, and H6 of the methane protons of N-acetyl glucosamine. An intense peak at 5.23 ppm is related to the H1 of N-acetyl glucosamine [44,45]. The structural characterization of thiolated chitosan by ^1^HNMR is displayed in Figure 2, containing a peak at 2.1 ppm that may corresponds to the protons of the newly attached side chain. The peak at 0.8 ppm corresponds to the –CH2-SH of the newly attached thiol group in thiolated chitosan, and a strong signal was detected at 4 ppm that may correspond to the remaining protons of the amine group (-NH_2_-) after the derivatization of chitosan. A strong peak at 5 ppm was associated with proton H1 of the hydroxyl group [46,47].

### 3.3. Fourier-Transform Infrared-Attenuated Total Reflectance (ATR-FTIR) Spectroscopy

**Figure 3:**
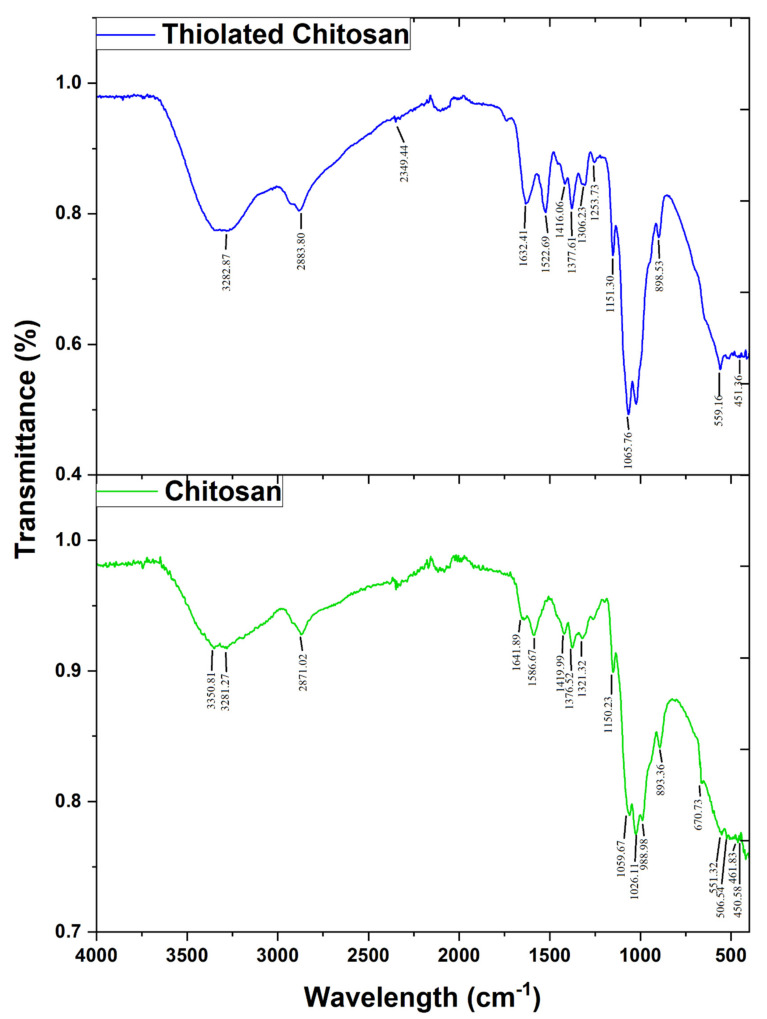
Fourier-transform infrared-attenuated total reflectance (ATR-FTIR) spectra of chitosan and thiolated chitosan synthesized by covalent bond formation between thioglycolic acid and chitosan via EDAC coupling.

Chitosan (CS) characteristic peaks are present at 3350–3281.27 cm^−1^, associated with amine NH symmetrical stretching vibrations, and overlapped by the broad absorption of the –OH group at 3300 cm^−1^ (Figure 3). The presence of residual N-acetyl groups was found in bands at about 1641.89 cm^−1^ (C=O stretching of amide-I) and 1376.52 cm^−1^ (C-N stretching of amide-III), and a small band at 1586.67 cm^−1^ that corresponds to the N-H bending of amide II [48]. The CH_2_ bending and CH_3_ symmetrical deformations were confirmed by the presence of bands at around 1419.99 cm^−1^ and 1376.52 cm^−1^, respectively. Bands at 1641.89 cm^−1^ (N–H deformation), 2922 cm^−1^ and 2871.02 cm^−1^ (C–H stretch), 1150.23 cm^−1^ (asymmetric stretch of C-O-C bridge), 1586.67 cm^−1^ (NH bend), 1026 cm^−1^ (C–O stretch, primary hydroxyl group), 1059.67 cm^−1^ (C–O stretch, secondary hydroxyl group) are present, as reported in the literature [49,50]. The representative peaks appearing at 1316 cm^−1^ are due to –CH_3_ symmetrical deformation, C–O stretching vibrations from (C–O–C) are at 1201 cm^−1^ and 1071 cm^−1^ [51].

ATR-FTIR analysis was performed to confirm the coupling of thioglycolic acid (TGA) with CS (Figure 3). The peak observed at 1632.41 cm^−1^ (C = O stretching amide I) and the deformation of the signal observed at 3282.87 cm^−1^ (-NH stretching amide) proves the formation of the amide bond (C-NH). The peak at 1522.69 cm^−1^ was assigned to the amide II band (NH bend and CN stretching) in CS-TGA. The peak at 2883.80 cm^−1^ due to stretching proves the existence of thiols as terminal groups connected to the chitosan. The presence of the peaks at 1253.73 cm^−1^ (C-SH stretching) and 898.53 cm^−1^ (S-S bisulfide bond) in thiolated chitosan confirms the presence of the thiol band, since such peaks are not seen in pure chitosan FTIR spectra. The absorption band at 3332.98 cm^−1^ was for CS-TGA-OH. The band from 997.19 to 1065.76 cm^−1^ resulted from CO, and the 2883.80–2900 cm^−1^ band was due to aliphatic CH stretch. The weak peak at 2580.44 cm^−1^ corresponds to SH, confirming the conjugation between the primary amine of CS and the thioglycolic acid CS-TGA. TC has three characteristic peaks at 1243 cm^−1^, corresponding to the vibration of the C-S bond [29,52].

### 3.4. Differential Scanning Calorimetry (DSC)

**Figure 4:**
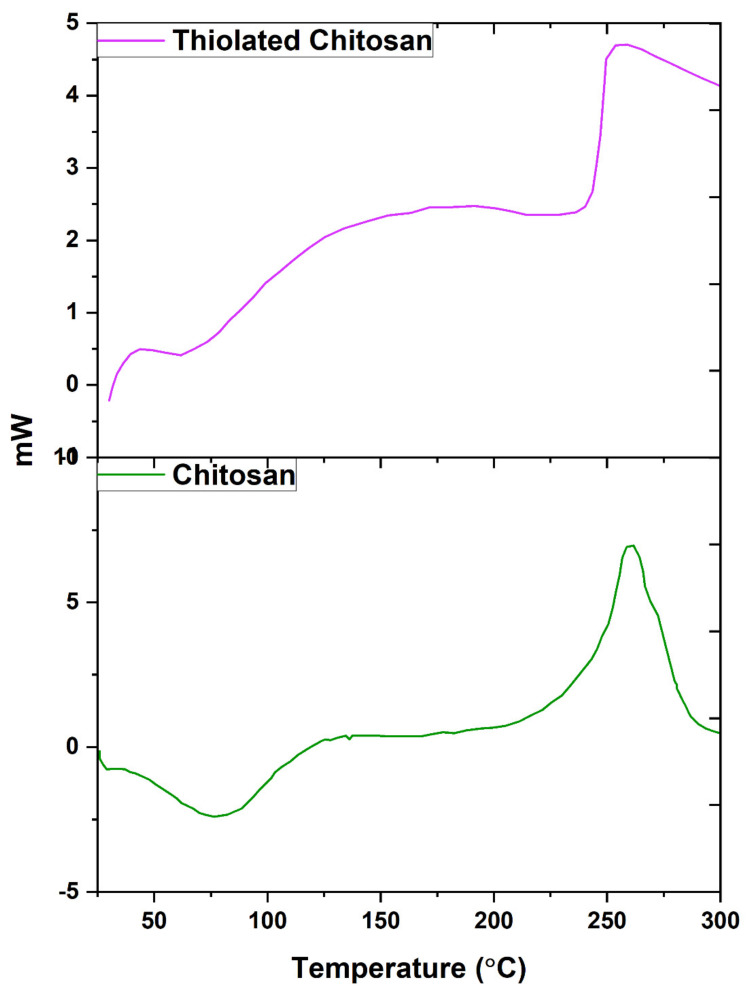
Differential scanning calorimetry thermogram of chitosan and thiolated chitosan synthesized by covalent bond formation between thioglycolic acid and chitosan via EDAC coupling.

The DSC thermogram of chitosan, shown in Figure 4, indicates an endothermic peak between 33–124 °C with heat of enthalpy (DH) at −132.70 J/g and peak area of −570.60 mJ. The exothermic peak between 200–280 °C has a heat of enthalpy (DH) at 19.9068 J/g and a peak area of 55.292 mJ. The endothermic peak, also called dehydration temperature (TD), is assigned to the loss of water associated with the hydrophilic groups of chitosan. In a solid state, the chitosan polysaccharide has a disordered structure and a strong affinity for water; as a result, it can be easily hydrated. This peak suggested that chitosan was not completely dried and that there was still some water molecules bound to it that may not be removed during drying. The exothermic peak is assigned to the thermal degradation of chitosan (monomer dehydration, glycoside bond cleavage, dehydration of the saccharide rings, depolymerization, and decomposition of the acetyl and deacetylated units) [53].

After thiolation, the endothermic peak of thiolated chitosan was present between 43–93 °C with a heat of enthalpy (DH) −16.29 J/g and a peak area of −60.29 mJ. The broad exothermic peak was found around 250–300 °C with a heat of enthalpy (DH) 200.6942 J/g and a peak area of 230.80 mJ (Figure 4). These peaks show that the structure of chitosan has been changed due to the thiol modification and reduced crystalline nature of chitosan. TC is a hygroscopic material; due to the water loss during degradation, it has a broad exothermic peak. The exothermic peak shows the degradation of thiol groups in thiolated chitosan, dehydration of the saccharide rings, depolymerization, and decomposition of the acetyl and deacetylated units. TC degradation starts at higher temperature (250 °C) compared to chitosan due presence of the amide bond (NCO-CH_2_-SH) formed during thiolation. Our observations are consistent with the literature [30].

### 3.5. X-ray Diffraction (XRD)

**Figure 5:**
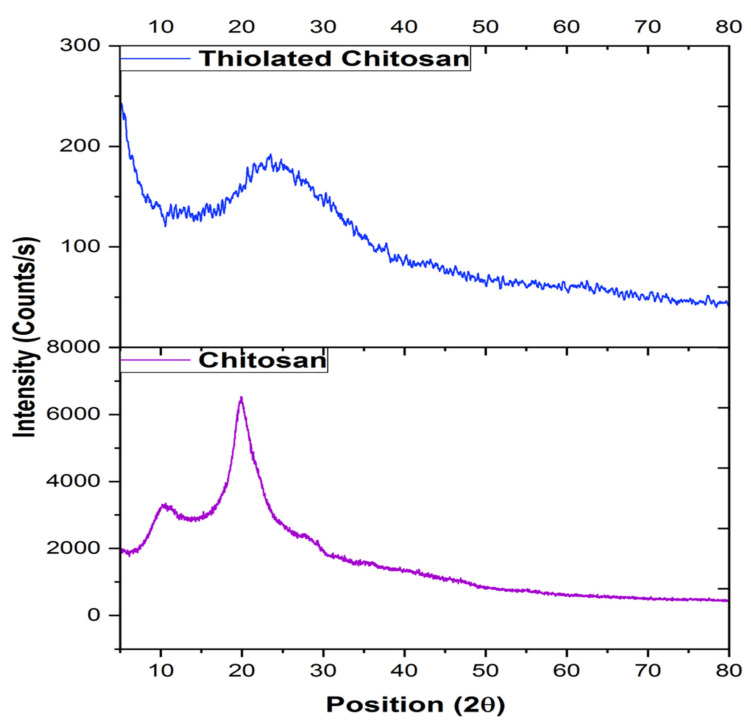
X-ray Diffraction spectra of chitosan and thiolated chitosan synthesized by covalent bond formation between thioglycolic acid and chitosan via EDAC coupling.

In the spectrum (Figure 5), chitosan exhibited two sharp peaks at 2θ = 10° and 2θ = 25°, indicating the regular crystal lattice of chitosan (crystal form I and crystal from II, respectively) [54]. As compared to the XRD pattern of chitosan [55], there was a noticeable change in the peaks of thiolated chitosan (Figure 5), suggesting the assimilation of –SH– groups in the chemical structure of chitosan and indicating a change in the crystallinity, which might be due to alterations in the inter polymeric atomic density [30]. The characteristic peak of chitosan was decreased and shifted to 2θ = 23.5° in thiolated chitosan, that is due to the reduction in number of free amino groups and reduction in intra molecular and intermolecular hydrogen bonding, which led to the lower crystallinity of TC [44].

### 3.6. Fabrication of Levosulpiride-Loaded Thiolated Chitosan Microneedle Patch (LS-TC-MNP)

Most of the water-soluble polymers (polysaccharides) are mechanically weaker than silicon or other non-soluble materials. Chitosan (CS) has exciting potential for drug delivery and biomedical applications. However, its use is, in some cases, limited due to its solubility issues, pH-dependent changes in electrostatic charge, and low mucosal adhesion. In order to overcome these limitations and make CS a more tunable polymer, various chemical modifications have been reported, such as quaternized CS, amphiphilic CS, steroidal/fatty acid derivative CS, aryl/alkyl derivative CS, and thiolated CS. These grafted polymers have been used in a variety of applications, including drug delivery, tissue engineering, in the textile industry, water purification, and many other biomedical applications [26]. Hence, thiolated chitosan (TC) has excellent mechanical strength and high water uptake ability, which may be due to the thiol moieties; therefore, thiolated chitosan (TC) was selected as the manufacturing material for the microneedle patch (MNP). Thiolated chitosan was also chosen due to its well-known biocompatibility and biodegradability [25]. In this study, a levosulpiride-loaded thiolated chitosan microneedle patch (LS-TC-MNP) was fabricated that could efficiently and sustainably deliver LS into the blood stream. The process of fabrication of microneedles should be moderate and well-controlled to avoid degradation of the LS. Microneedle patches were fabricated by using different concentrations of thiolated chitosan (Table 1). Different MNPs loaded with LS were successfully fabricated. Among the fabricated MN patches (LS-TC-MNP-1 to LS-TC-MNP-5), the MN patch fabricated by 3% TC (LS-TC-MNP-3) appears to be the best MN patch. The obtained LS-TC-MNP-3 has 100 needles, each with a length of 575 μm and a base diameter of 200 µm. Under microscopic observation, the needles appeared with pointed ends. The LS-TC-MNP-3 fabricated from TC (3%) showed the best performance in terms of ease of manufacturing, sharpness of needle, and tensile strength. Lower concentrations of TC solution result in bubble formation in the MNP during drying, alsoit is difficult to pour higher concentrations of TC into the mold to fill the pores.

### 3.7. Characterization of Microneedle Patch

Simply filling the microneedle molds with chitosan solution and then drying will not produce solid, strong microneedles. This can be attributed to void structure formation in the microneedle array after evaporation of water [25]. To overcome this issue, we used 3% TC to fill the mold twice using a two-step casting process. During the casting process, horizontal centrifugal force is utilized to fill the TC inside the microneedle mold. Characterization of LS-TC-MNP was performed by different techniques and tests as described below.

#### 3.7.1. Scanning Electron Microscopy Study

**Figure 6:**
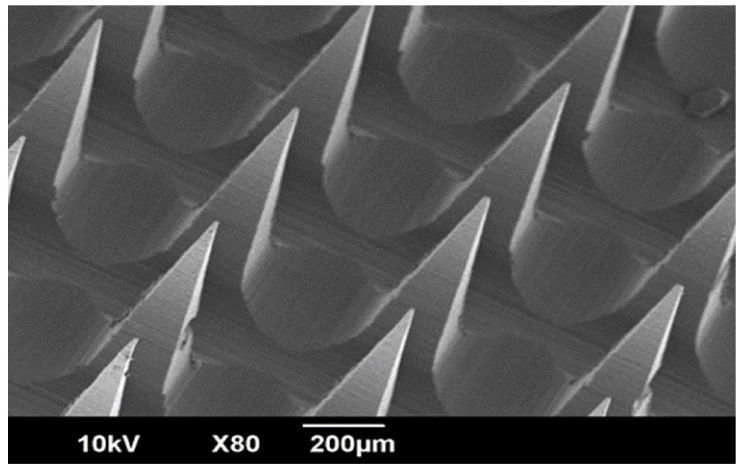
SEM image of LS-TC-MNP-3, showing polyhedral pyramidal-shaped microneedles with a smooth surface. The patch consists of 100 needles in 10 rows each with 10 needles. The length of the needle was 575 μm having sharp pointed end and base diameter of 200 µm.

The morphology of LS-TC-MNP-3 was analyzed by SEM. Figure 6 shows that LS-TC-MNP-3 has complete pyramidal-shaped microneedles, with a length of 575 µm, sharp pointed ends, and base diameter of 200 µm. The base and surface of needle was smooth, indicating that the whole LS-TC-MNP-3 was successfully fabricated. The SEM analysis of LS-TC-MNP-3 confirmed the existence of polyhedral pyramidal-shaped microneedles with sharp pointed ends and smooth surfaces. The patch consists of 100 needles in 10 rows each with 10 needles.

#### 3.7.2. Fourier-Transform Infrared-Attenuated Total Reflectance (ATR-FTIR) Spectroscopy

**Figure 7:**
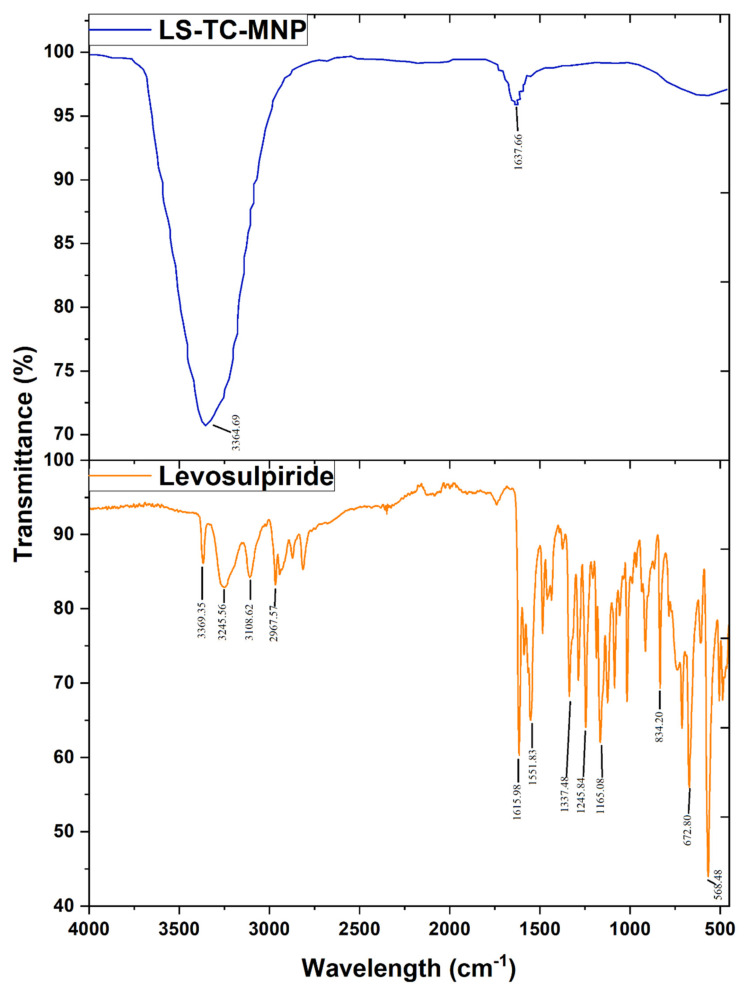
Fourier-transform infrared-attenuated total reflectance (ATR-FTIR) spectra of levosulpiride and levosulpiride-loaded thiolated chitosan microneedle patch.

The ATR-FTIR spectrum of levosulpiride shown in Figure 7. It shows the characteristic peaks of levosulpiride in the regions of 3369.35, 3245.56, and 3108.62 cm^−1^, corresponding to the N−H of the sulfonamide, amide, and aromatic groups, respectively. The bands at 2966.44–2814.12 cm^−1^ represented the C−H of the methylene and methyl groups. The band at 1615.98 cm^−1^ was for the C=O of the amide group. Skeletal stretching of the benzene ring was seen at 1588.07 cm^−1^. The C−O of the methoxy group was seen at 1245.84 and 1165.49 cm^−1^. The absorption bands at 1551.97, 1337.48, and 834.20 cm^−1^ were assigned to N−H, SO_2_, and C−H, respectively [56,57]. After the fabrication of LS-loaded TC-MNP, there was no interaction among the ingredient and drug, as shown in Figure 7.

#### 3.7.3. Tensile Strength

**Figure 8:**
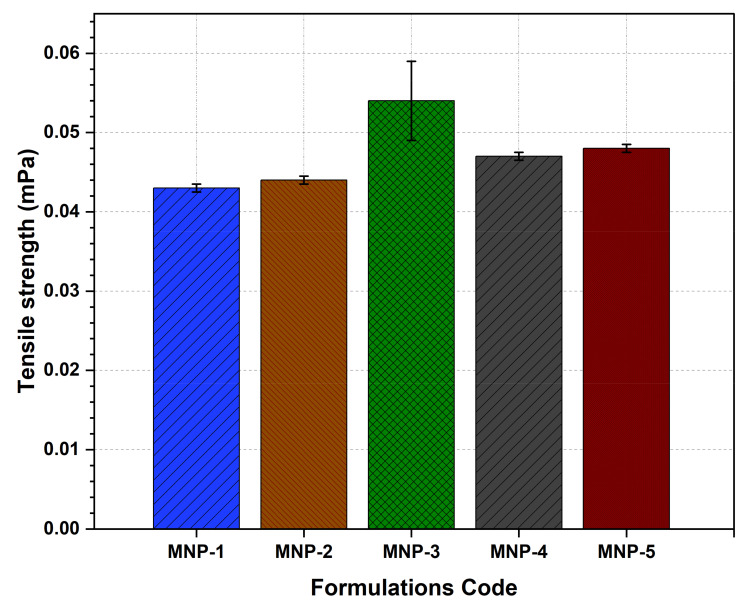
Evaluation of tensile strength of five different formulations of LS-TC-MNP fabricated with different concentrations of thiolated chitosan.

The tensile strength of LS-TC-MNP is shown in Figure 8. The LS-TC-MNP-3 with 3% TC showed a significant (*p*-value < 0.042) tensile strength of 0.052 mPa. The average tensile strength of LS-TC-MNP-1, LS-TC-MNP-2, LS-TC-MNP-4 LS-TC-MNP-5 were from 0.043 ± 0.038 to 0.047 ± 0.027 mPa. The tensile strength and percentage elongation of MNP play an important role in the complete removal of the dry patch from the mold. In addition, tensile strength and percentage elongation play an important role in handling and application on the skin [58]. Due to the presence of disulfide bonds, the final LS-TC-MNP-3 with 3% TC exhibits good tensile strength and reasonable percentage elongation due to the attachment of thiol groups, which may also increase the swelling properties of the polymer. An essential feature of polymer microneedles is the adequate mechanical strength for insertion into the skin. Factors affecting the mechanical strength of microneedles include material composition, geometry, and aspect ratio [25,36,59,60].

#### 3.7.4. Moisture Content

**Figure 9:**
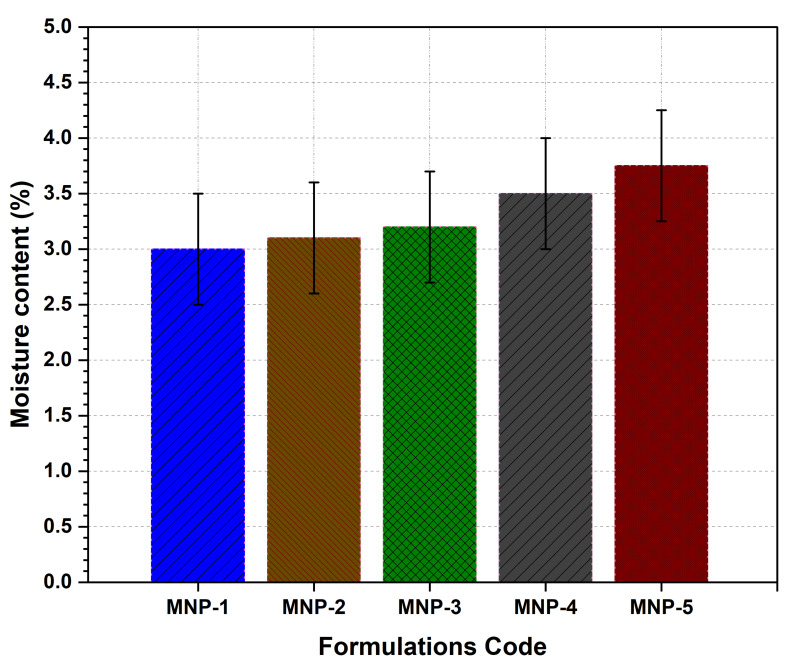
Evaluation of moisture content of five different formulations of LS-TC-MNP fabricated with different concentrations of thiolated chitosan.

The moisture content of the LS-TC-MNP was found to be 3.1 to 3.4% (Figure 9). The presence of moisture in the formulation or environment can adversely affect water-soluble polymers. TC has only a few OH groups as compared with other water-soluble polymers, such as hyaluronic acid and polyvinyl alcohol; because of its low water absorption rate (8.0%), it can maintain its mechanical strength and needle shape, even when the relative humidity is high (80%) [61]. The moisture content of LS-TC-MNP-3 has a significant amount of 3.2% (*p*-value < 0.0407), just enough to retain the mechanical properties of the MNP.

#### 3.7.5. Patch Thickness

**Figure 10:**
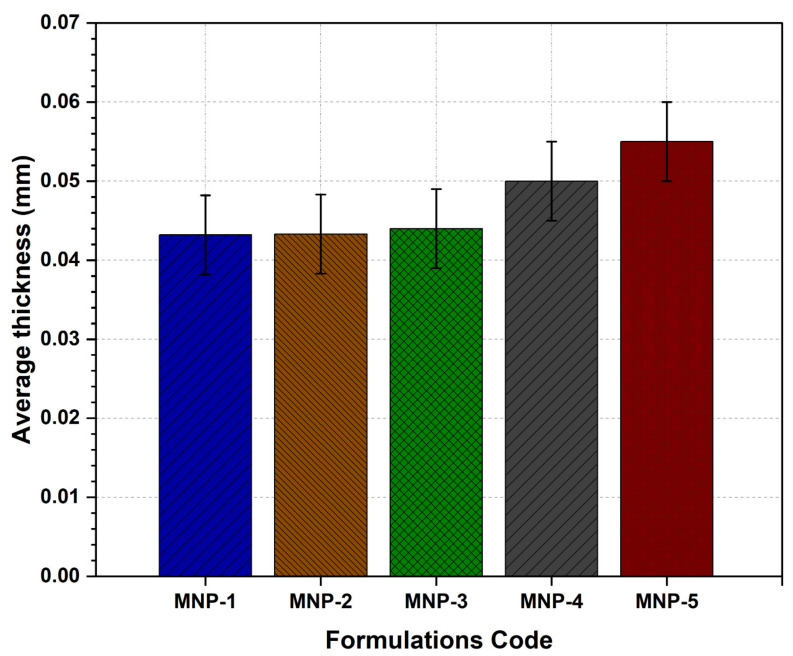
Evaluation of average thickness of five different formulations of LS-TC-MNP fabricated with different concentrations of thiolated chitosan.

The thickness of the resulting LS-TC-MNP is shown in Figure 10, and it was from 0.044 ± 0.0043 to 0.045 ± 0.0030 mm. The results show that the change in thickness was non-significant (*p* > 0.05), indicating homogeneity during mold filling and patch fabrication. These results are consistent with previous studies [62]. The thickness of the LS-TC-MNP was measured at various points with a micrometer. This is important to find out the uniformity of MNP thickness because it is directly related to the accuracy of the dosage in the patch. The MNP showed good uniformity, indicating the uniformity of drug loaded without loss of ingredients during fabrication.

#### 3.7.6. Percentage Elongation

**Figure 11:**
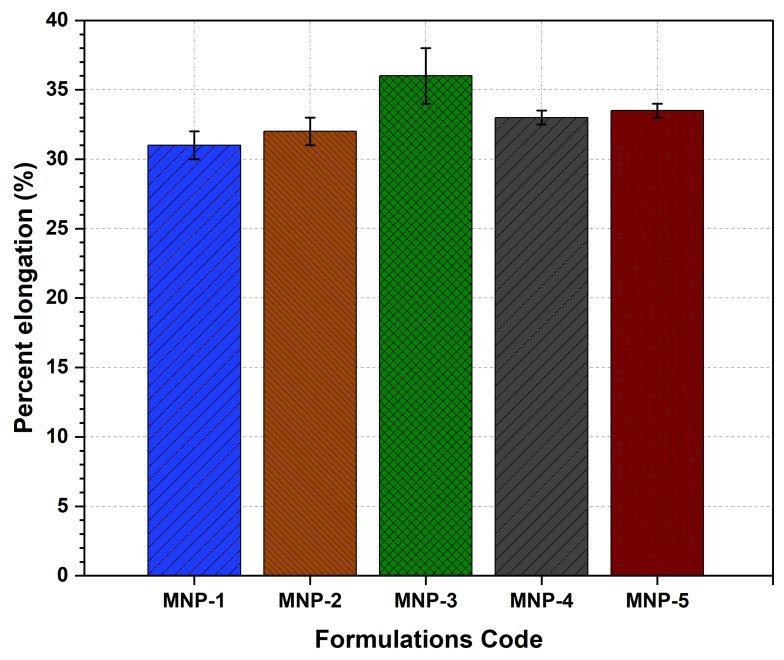
Evaluation of percentage elongation of five different formulations of LS-TC-MNP fabricated by different concentrations of thiolated chitosan.

An auto tensile tester was used to measure the percentage elongation of LS-TC-MNP. The results shown in Figure 11 indicate that the LS-TC-MNP-3 formulation has significant (*p*-value < 0.0284) elongation of 36 ± 4.4%.

#### 3.7.7. Penetration Ability

**Figure 12:**
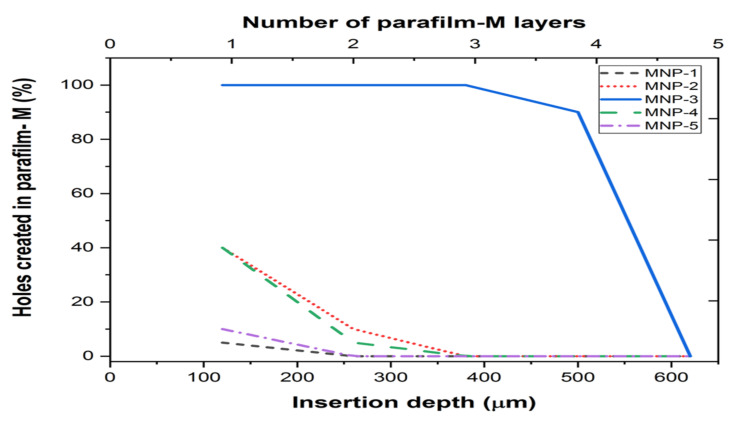
Measurement of the insertion depth of LS-TC-MNPs and percentage of hole formation in parafilm-M to study the penetration ability of microneedle patches.

LS-TC-MNP insertion studies were performed on parafilm-M to check the penetration ability. The results of the modified parafilm-M test are shown in Figure 12. The outcomes of the test were: LS-TC-MNP-3 could pierce first four layers of parafilm-M, reaching a depth of 500 µm. The number of holes was counted under microscope and 100 holes were found in the parafilm-M, confirming that the needles were intact and retained their tips, thereby forming 100 holes. The needles were also examined microscopically after removal, and no deformation was observed.

**Figure 13:**
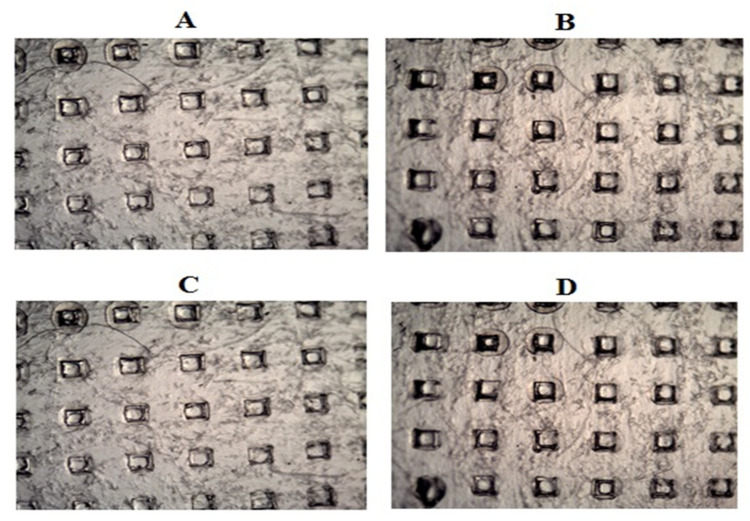
Microscopic image of parafilm-M layers: (**A**) first layer, (**B**) second layer, (**C**) third layer, and (**D**) fourth layer after insertion of LS-TC-MNP-3 in order to study the penetration ability of LS-TC-MNP-3.

LS-TC-MNP-3 needles could pierce to a depth of 500 µm, which is equivalent to 87% of the total length of the microneedles (575 µm). More than 95% of the needles retained their shape after removal from skin. Some needles lost their tips and were found in the parafilm-M. For LS-TC-MNP-1, LS-TC-MNP-2, LS-TC-MNP-4, and LS-TC-MNP-5 the percentage of intact needles was 0%, 20%, 18%, and 0%, respectively. The fragile nature of the needles indicates their brittleness due to the higher concentration of polymer that cannot withstand pressure. Similarly, at lower concentrations, the lower mechanical strength of the polymer leads to greater needle damage. It is known that the stratum corneum mainly acts as a major barrier to drug permeation through the skin. Herein, we showed that the LS-TC-MNP-3 can provide sufficient deep skin insertions, enhancing the pharmacological effects through enhanced LS bioavailability. The microscopic images of parafilm-M after the LS-TC-MNP-3 penetration study are shown in Figure 13A–D.

#### 3.7.8. Drug-Loading Efficiency

The drug-loading efficiency of all LS-TC-MNPs was between 38.46 ± 1.67 and 99 ± 1%. Lower concentrations of TC solution produce bubbles in the MNP after drying, and it is difficult to pour higher concentrations of TC into the mold and fill the pores of the mold. LS-TC-MNP-3 had a maximum loading efficiency of 99 ± 1% and a loading content of 20%. The TC 3% solution can easily fill the mold and the resultant LS-TC-MNP-3 contained full microneedles, giving it a larger capacity to hold the drug. Thiolated chitosan concentration, MN geometry, the number of microneedles, and the height of the needles in the array will affect the drug-loading capacity. In addition, the polyhedral shape has a longer length and a higher loading capacity compared to cylindrical microneedles [63].

#### 3.7.9. In-Vitro Drug Release Studies

**Figure 14:**
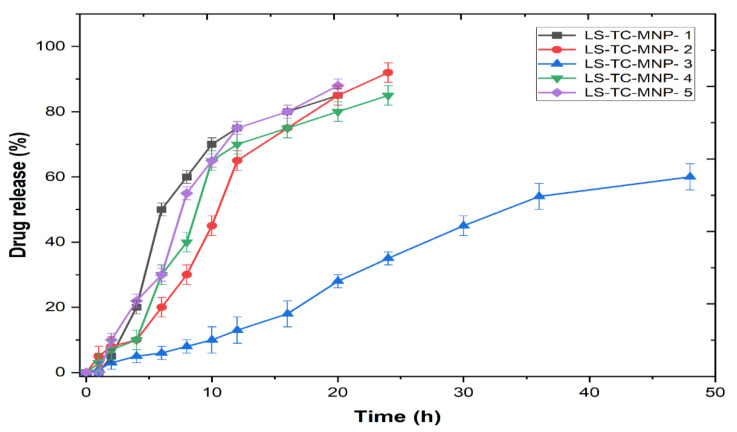
Graph showing the percentage drug release vs. time, from the five LS-TC-MNPs fabricated with different concentrations of thiolated chitosan (mean ±, n = 3).

The release curve showed sustained release up to 48 h and the maximum release was found to be 60% (Figure 14). The mathematical modeling results of the release data are shown in Table 2. Based on the value of R^2^, the LS-TC-MNP-3 follows the Korsmeyer–Peppas model. The value of n was 1.214, indicating that the release was super fall II.

The drug release mechanisms of biodegradable polymer-based formulations are divided into four categories: passive diffusion, matrix degradation, osmotic pumping, and controlled swelling [64]. In a controlled swelling-based system, the penetration of the solvent into the matrix can control the release rate. This is usually much slower than the diffusion of the drug [65]. The diffusion from swollen matrices is principally responsible for drug release; matrix degradation may also be effective for these systems [66]. It is believed that the LS-TC-MNP-3 in this study can provide the sustained release of LS over an extended period of time to overcome the shortcomings of multiple dosing regimen.

The mathematical modeling of the in-vitro release data provides information about the transport mechanisms that control the release of the drug from the delivery system. Zero-order release kinetics is required to achieve the desired sustained release mode in drug delivery [67]. This is mainly controlled by Fick’s law of diffusion under two mechanisms: (i) Fickian diffusion and non-Fickian diffusion [68]. As shown in Table 2, various models were applied to analyze the release kinetics of LS from LS-TC-MNP. Based on the R^2^ value, LS-TC-MNP-3 follows the Korsmeyer–Peppas model. The diffusion exponent, n, is an important indicator of various release mechanisms [69]. When the value of n is 0.5, the release mechanism follows Fickian diffusion (indicating diffusion-controlled drug release); 0.5 < n <1 (superposition of both phenomena) indicates an anomalous transport mechanism; n = 1 represents case II transport (representing relaxation/corrosion-controlled drug release); n > 1 represent super case II transport (representing drug release controlled by cross-linked polymer relaxation). In this study, the diffusion exponent (n) of the Korsmeyer–Peppas model showed that the release of LS was super fall II [68].

### 3.8. Ex-Vivo Permeation Study

**Figure 15:**
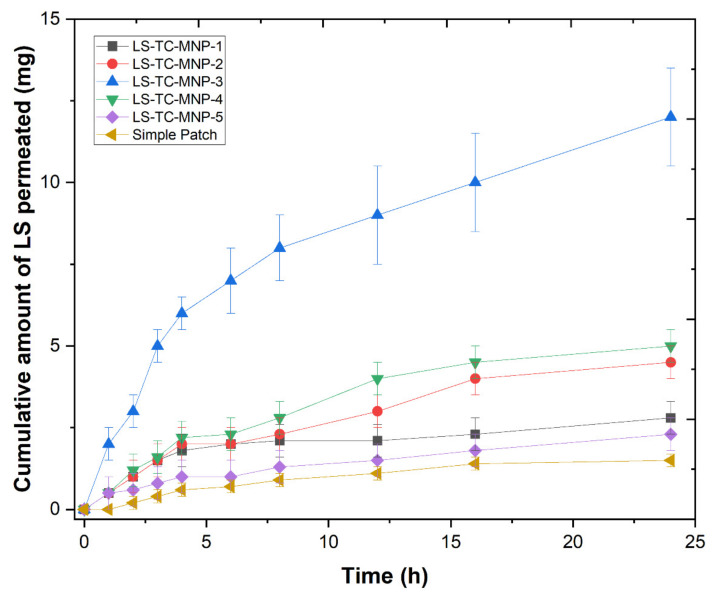
Graph showing cumulative amount of drug permeated vs. time through mice skin from the five LS-TC-MNPs fabricated with different concentrations of thiolated chitosan (means±, n = 3).

The results of the ex-vivo permeation study is shown in Figure 15. The results show that LS permeation from LS-TC-MNP-3 was successful, with nearly 12 mg/cm^2^ permeating through the mouse skin (24 h). The LS-TC-MNP-3 was not completely dissolved until 24 h. Thiolated chitosan (TC) is designated as a “thiomer”; these are widely used in non-invasive drug delivery [70]. TC has excellent potential for the control and maintenance of pH-dependent drug release [71]. TC has a higher degree of swelling under acidic pH values, ranging from 2 to 5, and a low degree of swelling under highly alkaline pH [72]. The pH of the skin surface is weakly acidic, between 4.5 and 5.5, and may vary with wounds and skin diseases. This acidic pH is a prerequisite for maintaining skin integrity, permeability, and homeostasis. The pH of the deep layers of the skin is close to neutral pH (7.4) to maintain natural compatibility with blood and body fluids [73,74]. This neutral pH limits the swelling behavior of TC. Therefore, after the insertion of LS-TC-MNP-3 (at neutral pH) in the deeper skin, the desired control release is expected.

It is very important to increase the permeability of the stratum corneum with LS-TC-MNP. LS-TC-MNP-3 (25 mg loading of LS) can significantly improve the permeation of LS through the skin within 24 h (*p* < 0.05). The micropores created by LS-TC-MNP-3 promote more effective transport (about 12 mg/cm^2^) of LS to deeper skin layers and ultimately to the blood vessels. The ex-vivo permeation through LS-TC-MNP-1, LS-TC-MNP-2, LS-TC-MNP-4, and LS-TC-MNP-5 was below 5 mg mg/cm^2^. The reason for low permeation is the shortness in the length of microneedles in the microneedle patch. During the formulation of microneedles with different concentrations of thiolated chitosan, the concentration of thiolated chitosan was increased in increments of 1, 2, 3, 4, 5%. When the concentration of thiolated chitosan was very low or high, the process of filling the microneedle mold was incomplete and resultant microneedle patch had bubbles, short and broken microneedles, fewer microneedles in the microneedles patch, and incomplete microneedle patch formation. All these shortcomings will result in improper insertion of the microneedles into the skin, meaning that the microneedle patch cannot properly deliver the drug. As the number and length of microneedles is shorter there will be lower drug loading, lower drug release, and lower permeation of the LS through LS-TC-MNP. LS-TC-MNP-3 formulated with 3% thiolated chitosan contains fully formed microneedles of 575 µm in length, a total 100 microneedles with no broken microneedles. LS-TC-MNP-3 formed needles of 575 µm in length, meaning that they cross the stratum corneum, epidermis, and viable epidermis, and reaches the dermis containing blood vessels. Hence, increased permeation was achieved with LS-TC-MNP-3. The presence of thiol groups significantly controls the absorption of water by LS-TC-MNP-3, which leads to moderate swelling and higher viscosity, thereby achieving a lasting effect in a longer time. The amount of the drug in the skin tissue was also quantified; 3.4% of the drug was found in the skin tissue, indicating that the total permeated amount of LS was 15 mg/cm^2^ (61.6%) from LS-TC-MNP-3.

### 3.9. Skin Distribution Study of LS-TC-MNP

The amount of LS in the skin was quantified by extraction of drug from the skin tissue. The results showed that 1.1 ± 0.6%, 2.3 ± 1.8%, 3.4 ± 2.4%, 1.9 ± 1.6%, 0.9 ± 0.9% of LS was present in the skin tissues after delivery through, LS-TC-MNP-1, LS-TC-MNP-2, LS-TC-MNP-3, LS-TC-MNP-4, LS-TC-MNP-5 respectively. The results show that a lower percentage of LS was delivered by LS-TC-MNP-1, LS-TC-MNP-2, LS-TC-MNP-4, and LS-TC-MNP-5 to the skin, due to fragile, broken, and incomplete microneedles that could not penetrate the skin. When compared to all LS-TC-MNPs, the LS delivered by LS-TC-MNP-3 was significant (*p*-value < 0.0376), 3.4 ± 2.4%, as a result of the good penetration and slow dissolution of microneedles in the skin.

### 3.10. In Vivo Tolerance and Safety Studies

One hour after the removal of LS-TC-MNP-3, the erythema score was 1, but there was no edema. However, the erythema was improved within 24 h, the erythema level was 0, and the skin had completely recovered from the redness after 48 h. The primary irritation index of the animals treated with LS-TC-MNP-3 was 2.3, indicating the medium value (because the PII of the medium value is between 2.0 and 4.9). An in vivo biocompatibility study for skin was performed to measure the skin irritation potential. Compared with the control group, the LS-TC-MNP-3 group displayed mild or moderate irritation at 1 h after LS-TC-MNP-3 removal. These symptoms may be caused by a higher number of needles, which may be parallel to the increase in the number of pores per unit area of the skin [75,76]. However, these local skin reactions disappeared in mice and no erythema was observed after 6 h. Therefore, these results indicate that the material and formulation are safe for skin application.

### 3.11. In-Vivo Study

**Figure 16:**
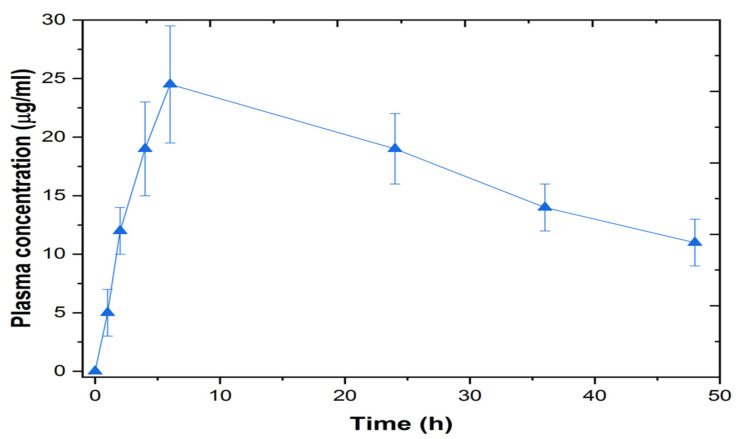
Graph showing the plasma concentration–time profile of LS-TC-MNP-3 fabricated with thiolated chitosan (applied on the back of the mouse) (Mean ± SD, n = 4).

The in-vitro and ex-vivo studies indicate that the LS-TC-MNP-3 had enhanced in-vitro sustained release of 60% and ex vivo permeation of 61.6%. Therefore, LS-TC-MNP-3 was selected for in vivo studies. In the transdermal treated group, one LS-TC-MNP-3 was applied on the back of each mouse. As shown in the graph of the plasma profile (Figure 16), LS was detected in mice plasma at a concentration of 5 ± 0.51 µg/mL at 1 h of the administration of LS-TC-MNP-3. The microneedles are made up of thiolated chitosan; after the insertion of LS-TC-MNP-3 into the skin, the polymer absorbs the interstitial fluid from the skin. After absorption of the interstitial fluid, the polymer starts to swell, causing the slow diffusion of the drug into the blood.

As a result of absorption of interstitial fluid, the thiolated chitosan swells more and more, releasing the drug into the skin and bloodstream. Therefore, the concentration increased to 24.5 ± 1.57 µg/mL after six hours and then slightly decreased to 19 ± 1.69 µg/mL after 24 h.

**Figure 17:**
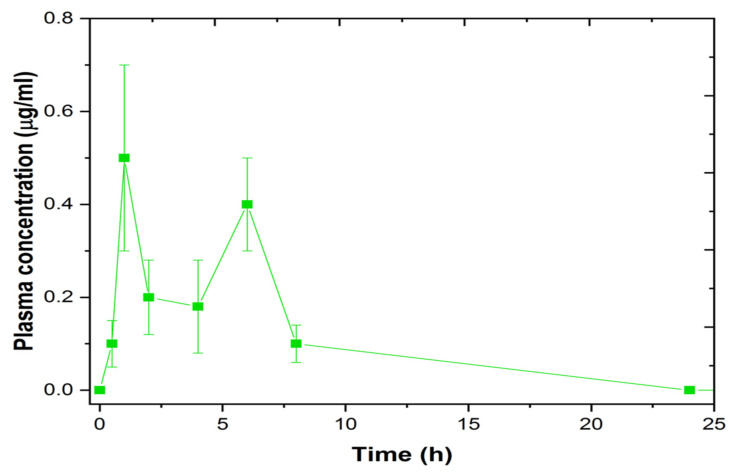
Graph showing the plasma concentration–time profile of levosulpiride after administration oral dispersion of levosulpiride to mice (at a dose of 200 mg/kg) (mean ± SD, n = 4).

The second group of mice received an oral dispersion of LS (200 mg/kg). As shown in the plasma profile graph (Figure 17), the concentration of LS in mice plasma was 0.3 ± 2.58 µg/mL after 1 h of oral feeding. The plasma concentration of LS is very low after oral administration because it is a BCS class IV drug. It has poor water solubility and poor permeability through the stomach lining. Therefore, less drug reaches the systemic circulation.

Due to low water solubility and low permeability, the desired therapeutic concentration of levosulpiride could not be achieved after oral administration. At 1 h, the LS concentration increased to a maximum of 0.5 ± 1.94 μg/mL. Then, after 2 and 4 h, the concentration decreased to 0.2 and 0.18 μg/mL, respectively. Then, at 6 h, the concentration of LS increased to 0.4 ± 1.37 μg/mL and then continuously decreased until 24 h (till the end point). These results are consistent with the previous studies, which reports that LS exhibits bi- or multi-phasic oral absorption peaks with low absorption, due to the different absorption sites and rates available for LS in the upper GIT [77]. Therefore, compared to the oral control group, the transdermal group has enhanced blood concentration and increased bioavailability of levosulpiride. No edema was observed at the application site.

As shown in the Table 3, the t_1/2_ of the LS-TC-MNP-3 increased to 11.04 ± 4.2 h, which is higher than that of the oral LS dispersion (5.24 ± 2.1 h). LS-TC-MNP-3 has a C_max_ of 24.5 ± 1.35 µg/mL, which is enhanced as compared to the oral dispersion with a C_max_ of 0.5 ± 0.2 µg/mL. AUC of LS-TC-MNP-3 was 986 ± 11.5 µg.hr/mL as compared to the oral dose. which was 3.2 ± 1.4 µg.hr/mL. All these pharmacokinetic results showed that the t_1/2_, C_max_, and AUC of LS-TC-MNP-3 was increased as compared to the oral dose. Thus, the transdermal treatment with LS-TC-MNP-3 will be a promising strategy for enhancing the bioavailability of levosulpiride and to overcome low oral bioavailability issues.

## 4. Conclusions

In the current study, levosulpiride was successfully delivered through transdermal route by using LS-TC-MNP-3. Chitosan was modified into thiolated chitosan (TC) by thioglycolic acid and EDAC coupling. Microneedle patches were fabricated by different concentrations of thiolated chitosan solution. Among all the prepared LS-TC-MNPs, LS-TC-MNP prepared with 3% TC (LS-TC-MNP-3) was proven to be the best MNP. The maximum drug loading efficiency of LS-TC-MNP-3 was 99 ± 1%. The in vitro release was sustained over 48 h, with a maximum release of 60%. The ex vivo results showed that the levosulpiride was successfully permeated by LS-TC-MNP-3, with nearly 61.6% permeated through the mouse skin (24 h). In vivo results showed the enhanced bioavailability of levosulpiride as compared to oral delivery. In conclusion, the levosulpiride-loaded thiolated chitosan microneedle patch was a stable, safe, and effective formulation for increasing the permeability and bioavailability of levosulpiride. As the field of microneedle technology progresses, it is important to consider different types of anti-schizophrenic drugs that can be delivered transdermally.

## Figures and Tables

**Table 1:** Different formulations of microneedle patch with their respective thiolated chitosan solution concentration and resultant levosulpiride-loaded thiolated chitosan microneedle patches with images, observation, and comments.

Formulation Code	Composition	MNP Obtained after Drying	Observation and Comment
LS-TC-MNP-1	1% thiolated chitosan solution	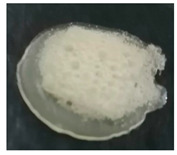	Resultant MNP was full of bubbles and brittle after dryning, no microneedles obtained.
LS-TC-MNP-2	2% thiolated chitosan solution	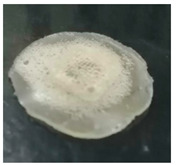	MNP formed with bubbles covering the surface of patch and no microneedles are visible
LS-TC-MNP-3	3% thiolated chitosan solution	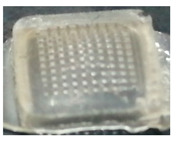	MNP formed with full length microneedles after drying.
LS-TC-MNP-4	4% thiolated chitosan solution	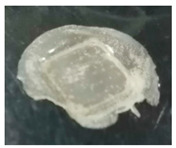	MNP obtained is brittle with no microneedles
LS-TC-MNP-5	5% thiolated chitosan solution	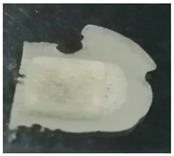	MNP is not obtained, due to very thick solution

**Table 2:** Table showing in-vitro release kinetic modeling of five LS-TC-MNPs fabricated with different concentrations of thiolated chitosan.

Formulations Code	Zero-Order		Korsmeyer–Peppas		Higuchi		Hixson–Crowell		First-Order	
	R^2^	K_o_	R^2^	N	R^2^	K_H_	R^2^	K_HC_	R^2^	K_1_
LS-TC-MNP-1	0.675	0.540	0.928	0.610	0.630	3.523	0.648	0.006	0.725	0.013
LS-TC-MNP-2	0.725	0.620	0.935	0.690	0.675	3.652	0.588	0.005	0.736	0.015
LS-TC-MNP-3	0.936	0.913	0.955	1.214	0.845	5.941	0.762	0.009	0.915	0.030
LS-TC-MNP-4	0.885	0.715	0.940	0.698	0.585	1.868	0.513	0.006	0.812	0.021
LS-TC-MNP-5	0.715	0.620	0.928	0.580	0.435	1.415	0.412	0.005	0.655	0.023

**Table 3:** Pharmacokinetic parameters of in vivo study up to 48 h after administration of oral dose and LS-TC-MNP-3 fabricated from thiolated chitosan (applied on the back of mice) (mean ± SD, n = 4).

Parameters with Units	Oral	LS-TC-MNP-3
t_1/2_ (h)	5.24 ± 2.1	11.04 ± 4.2
T_max_ (h)	2.1 ± 1.03	6.07 ± 3.4
C_max_ (µg/mL)	0.5 ± 0.2	24.5 ± 1.35
AUC (µg/mL·h)	3.2 ± 1.4	986 ± 11.5

## Data Availability

Data will be available upon request.
