# Correction: Habib et al. Thiolated Chitosan Microneedle Patch of Levosulpiride from Fabrication, Characterization to Bioavailability Enhancement Approach. *Polymers* 2022, *14*, 415

**DOI:** 10.3390/polym16233445

**Published:** 2024-12-09

**Authors:** Rukhshanda Habib, Abul Kalam Azad, Muhammad Akhlaq, Fakhria A. Al-Joufi, Gul Shahnaz, Hanan R. H. Mohamed, Muhammad Naeem, Abdulraheem S. A. Almalki, Junaid Asghar, Aamir Jalil, Mohamed M. Abdel-Daim

**Affiliations:** 1 Faculty of Pharmacy, Gomal University, Dera Ismail Khan 29050, Pakistan; rukhshandahabib.isb@gmail.com (R.H.); dr.akhlaq@gu.edu.pk (M.A.); junaid.asghar@gu.edu.pk (J.A.); 2 Department of Pharmacology, University of Oxford, Mansfield Rd., Oxford OX1 3QT, UK; 3 Department of Pharmacy, Quaid-I-Azam University, Islamabad 45320, Pakistan; gshahnaz@qau.edu.pk; 4 Department of Biotechnology, Quaid-I-Azam University, Islamabad 45320, Pakistan; mnaeem@qau.edu.pk; 5 Pharmaceutical Technology Unit, Faculty of Pharmacy, AIMST University, Bedong 08100, Kedah, Malaysia; 6 Department of Pharmacology, College of Pharmacy, Jouf University, Skaka 72341, Saudi Arabia; faaljoufi@ju.edu.sa; 7 Zoology Department, Faculty of Science, Cairo University, Giza 12613, Egypt; hananeeyra@gmail.com; 8 Department of Chemistry, Faculty of Science, Taif University, P.O. Box 11099, Taif 21974, Saudi Arabia; almalki.a@tu.edu.sa; 9 Faculty of Pharmacy, Bahauddin Zakariya University, Multan 60800, Pakistan; aamirs.jalil@gmail.com; 10 Department of Pharmaceutical Sciences, Pharmacy Program, Batterjee Medical College, P.O. Box 6231, Jeddah 21442, Saudi Arabia; 11 Pharmacology Department, Faculty of Veterinary Medicine, Suez Canal University, Ismailia 41522, Egypt

In the original publication [1], it has come to our attention that there was a duplication in Figure 13 upon publication. It appears that two sets of identical subfigures of the microscopic images of parafilm-M layers have been mistakenly used in Figure 13. The corrected Figure 13 appears below. The authors apologize for any inconvenience caused and state that the scientific conclusions are unaffected. This correction was approved by the Academic Editor. The original publication has also been updated.



## References

[1] Habib R., Azad A.K., Akhlaq M., Al-Joufi F.A., Shahnaz G., Mohamed H.R.H., Naeem M., Almalki A.S.A., Asghar J., Jalil A. (2022). Thiolated Chitosan Microneedle Patch of Levosulpiride from Fabrication, Characterization to Bioavailability Enhancement Approach. Polymers.

